# Cancer burden in China: a Bayesian approach

**DOI:** 10.1186/1471-2407-13-458

**Published:** 2013-10-06

**Authors:** Wanqing Chen, Bruce K Armstrong, Rongshou Zheng, Siwei Zhang, Xueqin Yu, Mark Clements

**Affiliations:** 1 National Central Cancer Registry, Cancer Institute, Chinese Academy of Medical Sciences, No.17 Pan-Jia-Yuan South Lane, Chaoyang District, Beijing 100021, China; 2 Sydney School of Public Health, The University of Sydney, Sydney, NSW, Australia; 3 Cancer Council New South Wales, Sydney, Australia; 4 National Centre for Epidemiology and Population Health, Australian National University, Canberra, Australia; 5 Department of Medical Epidemiology and Biostatistics, Karolinska Institutet, Stockholm, Sweden

**Keywords:** Bayes Theorem, China, Incidence, Mortality, Neoplasm

## Abstract

**Background:**

Cancer is a serious health issue in China, but accurate national counts for cancer incidence are not currently available. Knowledge of the cancer burden is necessary for national cancer control planning. In this study, national death survey data and cancer registration data were used to calculate the cancer burden in China using a Bayesian approach.

**Methods:**

Cancer mortality and incidence rates for 2004–2005 were obtained from the National Cancer Registration database. The third National Death Survey (NDS), 2004–2005 database provided nationally representative cancer mortality rates. Bayesian modeling methods were used to estimate mortality to incidence (MI) ratios from the registry data and national incidence from the NDS for specific cancer types by age, sex and urban or rural location.

**Results:**

The total estimated incident cancer cases in 2005 were 2,956,300 (1,762,000 males, 1,194,300 females). World age standardized incidence rates were 236.2 per 100,000 in males and 168.9 per 100,000 in females in urban areas and 203.7 per 100,000 and 121.8 per 100,000 in rural areas.

**Conclusions:**

MI ratios are useful for estimating national cancer incidence in the absence of representative incidence or survival data. Bayesian methods provide a flexible framework for smoothing rates and representing statistical uncertainty in the MI ratios. Expansion of China’s cancer registration network to be more representative of the country would improve the accuracy of cancer burden estimates.

## Background

Cancer is a leading cause of death in China [1]. Social and economic changes and population aging contribute to rapid increases in morbidity and mortality for most cancers. Timely and accurate data on cancer are essential for effective cancer control. However; there are currently no national cancer incidence data and limited cancer mortality data in China [2,3]. These deficiencies limit the evidence available for national policy on cancer control. An accurate estimate of the whole cancer burden and major types of cancer in China would greatly assist policy development.

The National Cancer Registration Network covered 43 regions from 20 provinces, with 5.53% of the national population, in 2005 [4]. Although this network provides very important data for China, there are shortcomings. Most registries are located in the developed urban areas of eastern China or are in known high-risk areas for cancers of the esophagus, stomach, liver and nasopharynx. The unbalanced distribution means that the registries do not paint a national representative picture of the cancer burden. Moreover, a low rate of pathological diagnosis in some registries and under-ascertainment limit the quality of the cancer registration data [5]. Thus the true burden of cancer in China cannot be currently estimated using cancer registration data alone.

For many developed countries, national estimates of cancer incidence are calculated from national cancer registration, nationally representative sentinel cancer registries or by constructing cancer incidence using national cancer mortality and relative survival from sentinel cancer registries [6]. For most countries, however, cancer incidence registries are not representative, nor are good survival data available. Under such circumstances, cancer survival would be indirectly represented using mortality to incidence (MI) ratios from available cancer registries and applying them to nationally representative cancer mortality statistics to calculate cancer incidence. The MI ratio is a good estimate of (1-survival) for good quality data [7] and the MI ratio has routinely been used to estimate incidence in the absence of representative incidence and survival data [8,9].

For incidence estimation, we adopted a Bayesian approach to estimate the MI ratios. Our statistical approach departed from that established by Jensen et al. and used by the International Agency for Research on Cancer (IARC) [8,9]. We modeled for the Poisson variability in incidence and mortality rates, smoothed across age using penalized splines, modeled for variation between cancer registries using random effects and estimated the statistical imprecision in the estimates of the national cancer incidence.

Our aims for this paper were to improve the methods for indirect estimation of incidence and to apply those methods to estimate cancer incidence in China using updated national mortality data and regional cancer registries data.

## Methods

### Mortality data

The Third National Death Survey (NDS) was carried out in 2004 and 2005 to better understand mortality rates and their trends in China. A total of 213 counties or districts were selected as the survey points, including 160 national Death Surveillance Points, which collected vital statistics for China, and 53 high-risk areas for cancer. The Death Surveillance Points were selected to represent the national population. They were based on counties and stratified by geographic regions, with sampling further stratified by urban or rural location and per capita gross domestic product. The survey was specifically designed to be nationally representative [10]. Our analysis included 158 Death Surveillance Points covering 142,660,482 person-years. Cancer site-specific mortality rates were calculated by age, sex, and urban or rural location. Two death surveillance points were excluded because of implausible mortality rates.

### Incidence data

The National Central Cancer Registry of China collected cancer incidence data from population-based cancer registries in China. We evaluated the quality of the cancer registry data before our analysis (see Additional file 1: Table S1). These quality control indicators for each registry suggest that the data quality was relatively high with: 62.06% morphologically verified (ranging from 21.80 to 90.74%); 0.66 for the MI ratio (ranging from 0.53 to 0.87); 1.50% of death certificate only registration (ranging from 0.00% to 11.43%); and 2.59% of unspecified cancer sites (ranging from 0.01% to 4.93%). Although there was variation of the indicators between registries, overall quality was acceptable. There were 32 cancer registries reporting cancer registration data for 2004 and 2005. The registries identified new cancer cases from all hospitals, community health centers, medical insurance and death registries (for cases only identified by death certification). Registries obtained information on cancer deaths from the death surveillance system, which collected death information from hospitals and the Civil Administration Bureau with available cremation reports. Population information was obtained from official registration records. For this study, the data on cancer site (coded using ICD-10), sex and age at diagnosis were retrieved from each cancer registry’s database. Age was divided into 19 subgroups, including 0 and 1–4 years, five year age groups from 5–9 years to 80–84 years, and 85 years or older. Cancer registry locations were classified as urban (prefecture-level cities, provincial capitals and municipalities directly under the Central Government) or rural (counties and county-level cities).

### Population data

Population data were obtained from the Statistics Bureau’s population-based 1% sampling survey. The estimated national population was 1,307,560,000 in 2005. There were 282,071,816 males and 280,048,184 females in urban areas and 378,907,152 males and 366,532,848 females in rural areas. Regional population data in 2005 were extracted by sex, age and urban or rural location.

### Statistical analysis

The statistical method used is a generalization of that used by Jensen et al. [8]. Whereas Jensen and colleagues modeled mortality as a Poisson regression with the log of incidence as an offset, we modeled both mortality and incidence rates as Poisson regressions with shared parameters linking the two regression models. Moreover, Jensen and colleagues fitted their models within a frequentist generalized linear models framework, while we fitted generalized linear mixed models within a Bayesian Markov chain Monte Carlo framework. We showed in Additional file 2 that the Poisson model used by Jensen and colleagues does not incorporate the uncertainty in incidence, and from a simulation sub-study, we showed that their model leads to under-coverage, with potential over-fitting during model selection. To briefly outline our approach, we estimated the national cancer incidence in 2005 by applying a set of age-, sex- and site-specific MI ratios (by rural or urban location), predicted from the modeling of the region-specific registries data, to the estimated 2005 national mortality data from the 3rd NDS.

Regression models were developed separately for each combination of sex and location (urban and rural). In the following, age groups were indexed by *i* = 1,…,19 and registries were indexed by *j*. We assumed that (a) registry incidence had a Poisson distribution with mean exp(α_*i*_ + u_*j*_) × (registry population), where α_*i*_ represented the log of the mean age-specific incidence rate and u_*j*_ represented random intercepts for the different registries; (b) registry mortality had a Poisson distribution with mean exp(α_*i*_ + u_*j*_ + β_*i*_ + v_*j*_) × (registry population), where β_*i*_ represented the log of the mean age-specific MI ratio and v_*j*_ represented random intercepts for the different registries and (c) NDS mortality had a Poisson distribution with mean exp(γ_*i*_) × (NDS population), where γ_*i*_ represented the log of the age-specific mortality rate for the NDS. We could then estimate the national incidence using exp(γ_*i*_-β_*i*_) × (national population).

The parameters α_*i*_, β_*i*_ and γ_*i*_ were smoothed using penalized splines. The means for α_*i*_ were defined by $\phi_{0}+\phi_{1}\frac{i-10}{19}+\sum_{k=1}^{6}Z_{\mathrm{ik}}\psi_{k}$ where: ∅_0_ and ∅_1_ were fixed effects (unknown parameters with normal prior distribution with mean 0 and variance 10^4^) for the constant and linear term; *Z*_*ik*_ was from the design matrix for O’Sullivan splines with knots indexed by *k* at ages 45, 55, 65 and 75 years, where the design matrix was calculated using R code written by Wand and Ormerod, and *Ψ*_*k*_ were random effects with normal prior distribution with mean 0 and variance σ_α_^2^. We assumed that the precision σ_α_^–2^ had an uninformative distribution. The parameters for β_*i*_ and γ_*i*_ were smoothed in a similar manner, with their own precision terms. The random intercepts u_*j*_ and v_*j*_ were assumed to have normal prior distribution with means 0 and variance σ_u_^2^ and σ_v_^2^, respectively. The inverse of the variance terms, also called the precision terms, were given Gamma (0.001,0.001) distribution. The models used a 25 000 burn-in and then sampled every 25th iteration for 25 000 iterations. The sampling properties based on graphical plots indicated adequate mixing for most parameters after a burn-in of approximately 5,000 and sampling for another 5,000. The choice of 25,000 for the burn-in and 25,000 for sampling was conservative to ensure good mixing for all of the sites. The 1000 samples were used to calculate 95% CIs for different combinations of parameters, including the numbers of incident cases, and age-specific and age-standardised rates. The model was implemented using WinBUGS, with model specification and data manipulation using R and the R2WinBUGS and BRUGS packages. R and WinBUGS code can be found in the Additional file 2.

SAS version 9.2 (SAS Institute, Cary, NC), R version 2.13.0 (http://www.r-project.org) and WinBUGS version 1.4.3 were used for the statistical analysis.

### Sensitivity analyses

Five sensitivity analyses were undertaken. Firstly, we implemented the model using the SAS procedure PROC MCMC, which implements a random walk Metropolis algorithm rather than the Gibbs sampler popularized by WinBUGS. The two models gave very similar age-specific predictions (not shown in Results). The SAS procedure required some effort to ensure good mixing of the parameters, including how to block the parameters for updating, the choice of update distribution and the method for constructing the initial covariance matrix. Secondly, we assessed whether the use of MI ratios based only on cancer registry data gave different results to MI ratios based on mortality data form NDS and incidence data from the fewer cancer registries (26 in all) that covered NDS areas. The ratio of NDS mortality to cancer incidence may be more valid for the prediction equation since we used the equation to predict national incidence from national mortality, but we also expected the ratios to be less precise since they were based on smaller populations. Third, we examined the effect on the estimates of removing cancer registries one at a time from the estimation of MI ratios. Fourth, we compared the analysis results for MI ratios, incidence rates and incident cases when MI ratios were constrained to be 1 (using a logit transform) or less and when they were unconstrained, using the WinBUGS package. When case fatality is high, mortality rates may be greater than contemporary incidence rates if (i) incidence is falling rapidly, (ii) incident cancer cases are under-ascertained, (iii) mortality is over-ascertained, or (iv) incident cancer cases are under-ascertained to a greater degree than contemporary cancer deaths. Fifth, we examined whether the estimates were sensitive to the exclusion of ages less than 20 years. Re-fitting the models with this restriction, we found that the estimated number of cases did not vary significantly between models that excluded ages less 20 years compared with estimates from models that included all ages. For all sites, the relative differences were less than plus or minus 3%.

We also sought to validate the incidence estimates internally by comparing registered incident cases, as recorded by the Shanghai and Qidong registries for 2005, with estimated incident cases calculated by multiplying real deaths data in the registry areas by the estimated MI ratios. These two registries had 16.9% and 0.9% of all incident cases in 2005 in the 32 registries. This approach follows the validation approach used by Jensen and colleagues when they estimated cancer incidence in the European Community from available cancer incidence data, MI ratios and mortality data [8].

We received an authorization to use the data for this analysis from the National Central Cancer Registry. No personal information (such as, name, ID number, home address and personal contact details) were included when the analysis data were extracted from the database. Given the highly aggregated form of the data extraction, an ethics approval was not required for this study.

## Results

### Mortality to incidence ratios for cancer in China

The MI ratio for all cancers was 0.74 in males and 0.61 in females (0.69 and 0.53 in urban areas and 0.79 and 0.71 in rural areas, respectively) (Table 1). Liver cancer had the highest MI ratio in males (0.93) and females (0.97). Breast cancer and cervical cancer had low MI ratios (0.26 and 0.30 respectively). The cancer type specific MI ratios in urban areas were lower than those in rural areas, except for lung cancer in both sexes and liver cancer in women. Credible intervals about the MI ratios of different cancers in either sex varied in width from less than 1.0% to 46.6% of the point estimates. MI ratios were most imprecise in rural areas.

**Table 1:** Estimated MI ratios by site, location and sex, China 2005

**Site**	**Overall**		**Urban**		**Rural**	
	**Male**	**Female**	**Male**	**Female**	**Male**	**Female**
	**MI (95% CI)**	**MI (95% CI)**	**MI (95% CI)**	**MI (95% CI)**	**MI (95% CI)**	**MI (95% CI)**
Nasopharynx	0.64 (0.57,0.72)	0.64 (0.52,0.78)	0.56 (0.48,0.68)	0.55 (0.39,0.80)	0.72 (0.62,0.82)	0.72 (0.59,0.88)
Esophagus	0.80 (0.76,0.84)	0.83 (0.78,0.90)	0.77 (0.71,0.83)	0.86 (0.76,1.03)	0.81 (0.77,0.87)	0.82 (0.76,0.89)
Stomach	0.72 (0.69,0.75)	0.80 (0.77,0.84)	0.71 (0.68,0.74)	0.76 (0.70,0.84)	0.73 (0.69,0.77)	0.83 (0.78,0.87)
Colorectal	0.47 (0.43,0.52)	0.47 (0.42,0.52)	0.42 (0.38,0.46)	0.43 (0.38,0.49)	0.55 (0.45,0.65)	0.53 (0.43,0.63)
Liver	0.93 (0.87,0.98)	0.97 (0.91,1.02)	0.91 (0.84,1.00)	0.98 (0.90,1.09)	0.94 (0.87,1.00)	0.96 (0.89,1.03)
Pancreas	0.91 (0.81,1.00)	0.92 (0.82,1.05)	0.91 (0.77,1.06)	0.93 (0.78,1.11)	0.91 (0.80,1.03)	0.93 (0.80,1.04)
Lung	0.87 (0.83,0.92)	0.88 (0.82,0.96)	0.89 (0.81,0.97)	0.90 (0.78,1.05)	0.85 (0.81,0.90)	0.87 (0.82,0.92)
Bone	0.85 (0.72,0.99)	0.87 (0.70,1.08)	0.68 (0.53,0.89)	0.74 (0.52,1.08)	1.03 (0.86,1.23)	1.02 (0.80,1.28)
Breast		0.26 (0.23,0.29)		0.20 (0.17,0.23)		0.38 (0.34,0.43)
Cervix		0.30 (0.25,0.37)		0.23 (0.19,0.31)		0.37 (0.27,0.51)
Ovary		0.38 (0.32,0.45)		0.35 (0.28,0.43)		0.48 (0.37,0.59)
Prostate	0.43 (0.37,0.50)		0.34 (0.29,0.41)		0.60 (0.46,0.77)	
CNS	0.73 (0.66,0.83)	0.65 (0.53,0.79)	0.64 (0.54,0.77)	0.55 (0.40,0.77)	0.86 (0.75,0.97)	0.79 (0.68,0.92)
Lymphoma	0.68 (0.53,0.86)	0.60 (0.47,0.77)	0.62 (0.43,0.95)	0.53 (0.38,0.78)	0.77 (0.62,0.90)	0.72 (0.57,0.88)
All sites	0.74 (0.71,0.77)	0.61 (0.57,0.66)	0.69 (0.63,0.74)	0.53 (0.45,0.59)	0.79 (0.76,0.82)	0.71 (0.66,0.76)

**Figure 1:**
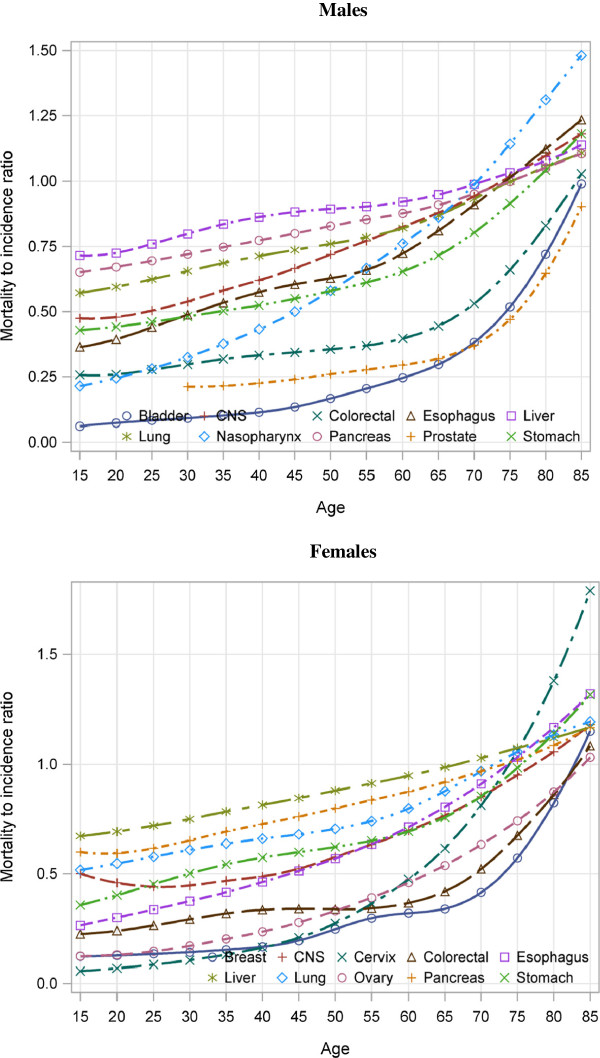
Modeled Age-specific MI Ratios for Major Cancers in 32 Cancer Registries in China 2005.

Modeled age-specific MI ratios for individual major cancer types were shown in Figure 1 for ages 15–19 years and older; estimated ratios in younger age groups were very imprecise because of small numbers. For all cancers in both sexes, there was an initial short plateau in the MI ratio followed by a rise to a peak in the oldest people. The pattern of change in MI with age varied among cancer types, but progressive increase with age was a more-or-less constant feature.

### Estimated cancer incidence rates in China

In urban areas, the estimated World age standardized incidence rates for all cancers were 236.2 per 100,000 for males and 168.9 per 100,000 for females. In urban areas, lung cancer had the highest incidence in males, while breast cancer was the most common cancer in females. In rural areas, the World age standardized incidence rates for all cancers were 203.7 per 100,000 for males and 121.8 per 100,000 for females. Stomach cancer was the most common cancer for both men and women in rural areas (Table 2). Credible intervals about the age-adjusted incidence rates for different cancers in either sex varied in width from 1.8% to 281.7% of the point estimates. The estimates were more precise in urban areas than in rural areas.

**Table 2:** **Estimated age-standardized cancer incidence rates per 100 000 by site, location and sex, China 2005**^**a**^

**Site**	**Overall**		**Urban**		**Rural**	
	**Male**	**Female**	**Male**	**Female**	**Male**	**Female**
	**Rate (95% CI)**	**Rate (95% CI)**	**Rate (95% CI)**	**Rate (95% CI)**	**Rate (95% CI)**	**Rate (95% CI)**
Nasopharynx	2.9 (2.5,3.5)	1.1 (0.8,1.6)	3.4 (2.5,4.4)	1.2 (0.8,2.1)	2.6 (2.1,3.3)	1.0 (0.7,1.5)
Esophagus	23.9 (22.1,25.8)	9.6 (8.6,10.6)	17.5 (15.4,19.9)	5.7 (4.6,7.0)	28.5 (25.9,31.5)	12.5 (11.1,14.0)
Stomach	42.1 (39.6,44.9)	17.4 (16.0,19.0)	37.1 (34.2,40.3)	15.3 (13.3,17.8)	45.6 (41.9,49.9)	18.9 (17.0,21.0)
Colorectal	16.6 (14.5,19.2)	11.8 (10.2,14.0)	22.4 (19.3,26.3)	15.6 (13.1,18.8)	12.3 (9.7,16.0)	8.9 (6.9,11.9)
Liver	36.8 (34.3,39.6)	12.7 (11.6,13.9)	33.2 (29.4,37.2)	10.7 (9.4,12.3)	39.5 (36.2,43.2)	14.2 (12.7,15.9)
Pancreas	3.2 (2.7,3.8)	2.2 (1.9,2.6)	4.6 (3.7,5.7)	3.4 (2.7,4.3)	2.1 (1.7,2.7)	1.4 (1.1,1.8)
Lung	45.6 (42.8,48.8)	19.7 (17.8,21.6)	53.0 (47.7,59.5)	23.1 (19.5,27.1)	40.2 (37.1,43.6)	17.0 (15.3,18.9)
Bone	2.3 (1.8,3.0)	1.5 (1.1,2.1)	2.9 (1.9,4.4)	1.8 (1.1,3.0)	1.8 (1.4,2.5)	1.2 (0.8,1.8)
Breast		20.4 (17.5,23.8)		31.2 (25.6,38.4)		11.5 (9.4,13.9)
Cervix		8.1 (6.3,10.5)		9.0 (6.4,12.6)		7.3 (5.0,10.5)
Ovary		3.6 (2.8,4.7)		6.0 (4.5,8.3)		1.6 (1.1,2.3)
Prostate	4.2 (3.4,5.5)		6.6 (5.0,8.7)		2.5 (1.7,3.9)	
CNS	4.7 (3.9,5.7)	3.9 (3.1,5.1)	6.1 (4.5,8.1)	5.1 (3.4,7.5)	3.6 (2.9,4.7)	2.9 (2.3,3.9)
Lymphoma	2.5 (1.9,3.4)	1.7 (1.2,2.4)	3.3 (2.1,5.2)	2.3 (1.5,3.7)	1.8 (1.3,2.5)	1.1 (0.8,1.8)
All sites	217.7 (206.7,228.9)	143.0 (132.1,156.8)	236.2 (216.2,258.3)	168.9 (148.8,198.0)	203.7 (192.4,215.1)	121.8 (111.7,133.4)

^a^Rates were directly age-standardized to the World population.

**Figure 2:**
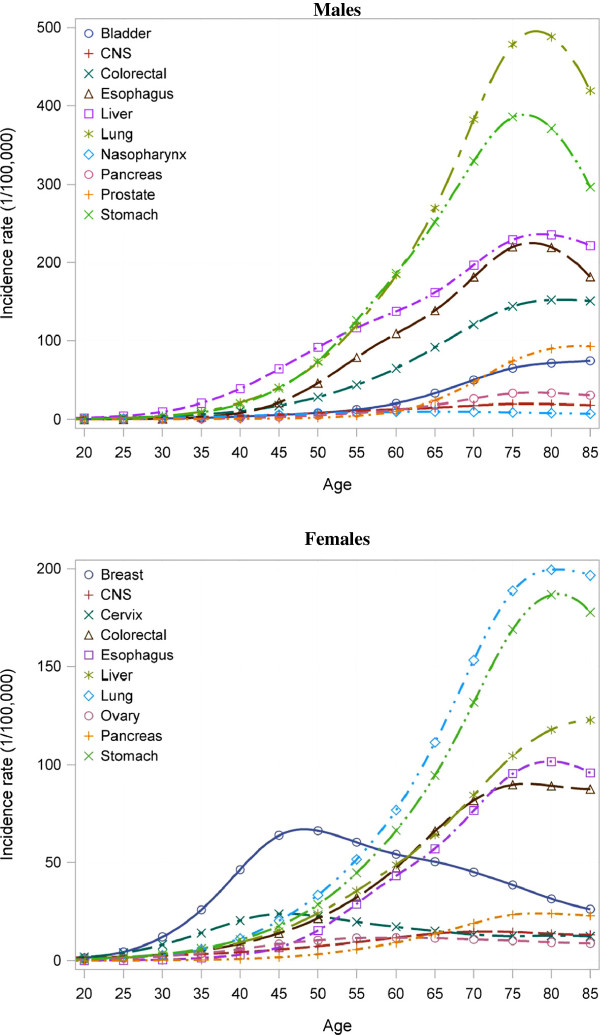
Estimated Age-specific Incidence Rates for Major Cancers in China, 2005.

Estimated age-specific incidence rates for the major cancer types were shown in Figure 2. In males, the rates were very low in subjects aged less than 40 years. After that age, they rose with increasing age in a typical pattern to a maximum at 80–84 or 85+ years of age. This pattern was similar in females except for breast and cervical cancers. Estimated incidence of those cancers began to rise at about 30–34 years of age, reaching a peak in women aged 40s to early 50s and then fell progressively, with a small upturn in incidence of cervical cancer in women in their 80s.

### Estimated incident cases of cancer in China in 2005

We estimated that there were 2,956,300 new cases of cancer diagnosed in China in 2005 (1,762,000 in males and 1,194,300 in females). Lung cancer was the most common cancer with an estimated 541,600 new cases in 2005 followed by stomach cancer (493,500), liver cancer (411,300), esophageal cancer (276,600), colorectal cancer (243,600) and female breast cancer (172,800) (Table 3). The precision of these estimates varied by cancer type, sex and location as expected due to the different size of the subpopulations (see Additional file 3: Table S2).

**Table 3:** Estimated numbers of incident cancer cases in 1000 s by site, and sex, China 2005

**Site**	**Overall**		**Urban**		**Rural**	
	**Male**	**Female**	**Male**	**Female**	**Male**	**Female**
	**Cases (95% CI)**	**Cases (95% CI)**	**Cases (95% CI)**	**Cases (95% CI)**	**Cases (95% CI)**	**Cases (95% CI)**
Nasopharynx	24.1 (21.6,27.3)	9.6 (7.7,12.0)	12.2 (9.8,14.6)	4.7 (3.1,6.7)	12.0 (10.3,14.1)	4.8 (3.9,6.0)
Esophagus	194.8 (185.4,204.9)	81.8 (75.4,87.5)	59.7 (55.3,64.8)	21.0 (17.6,24.3)	135.0 (126.8,143.8)	60.6 (55.7,65.6)
Stomach	343.9 (329.9,358.5)	149.6 (142.3,157.4)	128.8 (123.0,135.1)	56.9 (51.7,62.3)	214.9 (202.7,229.6)	92.7 (87.6,98.5)
Colorectal	134.9 (122.9,149.0)	99.7 (89.9,111.5)	77.3 (70.2,85.6)	57.1 (50.2,64.8)	57.4 (47.5,69.9)	42.4 (35.4,52.3)
Liver	303.3 (286.2,321.8)	108.0 (101.9,114.7)	117.9 (107.3,128.6)	39.7 (35.9,43.7)	185.4 (172.5,198.0)	68.4 (63.1,73.9)
Pancreas	26.1 (23.5,29.3)	19.2 (16.8,21.8)	15.9 (13.5,18.9)	12.4 (10.2,14.8)	10.2 (8.8,11.6)	6.7 (5.8,7.9)
Lung	373.0 (354.5,394.8)	168.6 (155.3,181.8)	183.1 (166.9,202.7)	85.4 (73.4,98.1)	190.1 (178.9,201.5)	83.0 (78.0,88.7)
Bone	17.7 (15.0,21.0)	11.7 (9.3,14.6)	9.4 (7.1,12.2)	6.3 (4.3,8.8)	8.3 (6.8,9.9)	5.4 (4.2,6.9)
Breast		172.8 (154.3,194.9)		119.2 (102.8,140.5)		53.5 (47.2,59.7)
Cervix		68.5 (56.1,84.0)		35.6 (27.0,45.5)		33.0 (24.5,44.9)
Ovary		29.9 (25.0,36.2)		22.5 (17.9,28.6)		7.3 (5.8,9.5)
Prostate	34.8 (29.6,40.4)		22.8 (18.7,27.4)		11.8 (9.1,15.7)	
CNS	35.6 (31.3,40.2)	30.5 (25.0,37.2)	19.8 (15.9,23.5)	17.6 (12.6,23.9)	15.8 (13.9,18.2)	12.8 (10.8,15.0)
Lymphoma	19.8 (15.5,25.3)	13.5 (10.4,17.4)	11.5 (7.5,16.6)	8.4 (5.7,12.1)	8.3 (6.9,10.2)	5.1 (4.0,6.5)
All sites	1762.0 (1686.9,1836.6)	1194.3 (1115.1,1295.7)	811.1 (751.4,878.5)	619.3 (551.2,716.6)	950.2 (907.8,987.1)	575.0 (534.8,619.3)

### Sensitivity analyses

The estimates of national incidence based on MI ratios calculated from cancer registries in NDS areas were similar to estimates based on MI ratios from all cancer registries for all cancers and most cancer sites. For cancers of the colon and rectum, nasopharynx, lung and stomach, and for lymphoma, the differences were less than 4.0% of the estimates of national incidence and approximately equally distributed between estimates greater than and less than those based on NDS areas. As expected, the intersection between the cancer registries and the NDS gave considerably fewer events, which led to imprecise estimates for several cancer sites. Point estimates for the predictions for some cancer sites varied appreciably under the sensitivity analysis, where malignant tumors of brain had 14.5% few predicted cases and bone had 8.3% more predicted cases.

Removing any registry from the estimation of the MI ratio, made only small changes to burden estimates. The effect on all cancers ranged from 0.01% to 1.22% of the total incidence estimates. Registries with very large populations did not strongly influence the estimates, for example, 0.41% from removal of Beijing registry and 0.59% Shanghai registry. Shenyang registry had the largest effect (1.22%), possibly because of its quite large population (more than 3 million) and comparatively high incidence rates of cancers with high MI ratios, such as lung cancer and liver cancer.

When we compared cancer burden calculated with MI ratio estimates constrained to 1 or less with that calculated with no constraint on the MI ratio, our primary analysis, the different for all cancers was 6% (95% CI: 6% to 14%) in males and 0% (95% CI: -9% to 7%) for females. For individual cancers, the two were different by not more than 8% in males and in females.

### Internal validation

When estimated national MI ratios were applied to Shanghai cancer mortality data the overall cancer incidence in Shanghai was estimated at 5.4% in males and 9.5% in females higher than its observed incidence. Similarly for Qidong, the estimated incidence was 2.3% less in males and 4.7% higher in females than the observed incidence.

## Discussion

We estimated a total of 2.96 million incident cases of cancer in 2005 in China by using data from 32 cancer registries and the third National Death Survey, and a Bayesian model. Among all cancers, lung, stomach, liver, esophageal, colorectal and female breast cancer were the most common cancers. The incidence of those cancers was higher in males than in females (except for breast cancer). For cancers of the esophagus, stomach and liver the incidence was higher in rural locations than in urban locations and for cancers of the lung, female breast and colorectum, the reverse patterns was observed. MI ratios were not consistently different between the sexes but, with few exceptions, were generally higher in rural than urban locations.

There have been two recent efforts to estimate cancer burden in China. First, the IARC estimated that there were 2.82 million new cancer cases and 1.96 million cancer deaths in China in 2008 in its GLOBOCAN project [9]. It estimated age, sex and site-specific MI ratios using 2003–2005 data from 36 Chinese cancer registries. The authors followed the model from Jensen et al. [8]. Younger age groups were combined and age was smoothed using polynomials up to order 5, requiring a model selection step using likelihood ratios [8,9,11]. National incidence rates for the rural and urban populations of three regions (East, Middle and West) were calculated from the products of the MI ratios and mortality rates from the NDS (2004–2005) and incident numbers were estimated by multiplying by the regional populations for 2008. These numbers were probably under-estimated for 2008 because of the use of mortality data for 2004–2005 and generally increasing mortality rates since then. In addition, the authors chose to stratify by both region and urban/rural location, which would result in only a few registries contributing to some strata. If the registries contributing to a stratum were not representative, then their MI ratios would poorly predict incidence in that stratum. Moreover, the predictions at younger and older ages were likely to be unstable or inaccurate, as polynomials are imprecise at data boundaries. No measures of uncertainty were presented.

Second, Ren and colleagues compared two methods to estimate cancer incidence in China using data and methods similar to those used in GLOBOCAN [12]. Their preferred method gave estimates of 2.58 million incident cancer cases and 1.79 million cancer deaths in 2005. For calculating the MI ratios under this method, smaller cancer registries were given more weight by dividing the numbers of incidence and mortality cases by the square root of the registry population. They did not quantify uncertainty; nor could they validly estimate uncertainty using the approach adopted, as an incident count divided by the square root of population does not have a Poisson distribution. Our estimate of 2.96 million was about 15% higher than that obtained by Ren el al using more conventional methods. One possible explanation for the difference in estimates is that we used a formal statistical model for the variation between cancer registries using random effects, while Ren and colleagues used arbitrary weights for each cancer registry.

Modeling within a Bayesian MCMC framework allowed for the ready calculation of credible intervals (CIs) for MI ratios, age-standardized rates and numbers of incident cancers. We were able to use an appropriate statistical model for the incidence and mortality rates, to flexibly smooth across age groups using random effects, and to estimate reliable age-specific estimates. Moreover, to take better account of variation between the cancer registries, we included registry-level random effects for cancer incidence and for MI ratios. Care may be required in the interpretation of the CIs, as the registry-level random effects may under-estimate the uncertainty associated with generalizing the MI ratios from the cancer registries to the national population. One strength of our approach is that we could provide a reasonable lower bound on the level of uncertainty for these estimates. The uncertainty was larger for rural areas, where less of the population was represented and cancer registrations tend to be less reliable. Uncertainty was also greater for younger and older age groups, where smaller numbers of cancers lead to wider CIs.

Some indication of the uncertainty due to imperfect measurement systems was given by the results of our sensitivity analysis on the source of the mortality data. Generally, use of NDS mortality instead of mortality recorded by cancer registries to estimate MI ratios produced incidence estimates that were within 4% of those obtained using only the registry data, which suggests good agreement between the two approaches in the recording of cancer mortality. There was, however, much greater variance than this for some cancers, which may reflect systematic error in one or other cancer mortality data collection. For the sensitivity analysis were the MI ratios were estimated from the overlap between cancer registries and the NDS, the national burden of bone cancer was 8% higher, which may, perhaps, reflect a greater tendency for the NDS to records deaths from secondary cancer in bone as death from primary cancer, while the national burden for brain cancer was 14.5% lower, which may indicate a tendency for the stigma attached to brain cancer to lead to under-recoding of deaths from it in the NDS. It is also important to note that the 32 cancer registries on which our reported national cancer incidence was based covered a little less than 5% of China’s population, and most were located in the east of China, which was more developed economically than the west of the country. Thus use of limited cancer registry data to estimate national burden will almost inevitably lead to some bias in the estimate. MI ratios by cancer registry are available in Additional file 4: Table S3. As more registries become available, it will be useful to investigate whether there is variation of burden in China across an east–west axis and across a north–south axis.

On a technical note, we assumed that the counts for incidence and mortality are statistically independent, albeit with tightly linked means; this is not strictly true, as we expect that deaths today will be a result of incident cancers over the preceding years. However, formal statistical modeling for this autoregressive relationship, such as by use of MIAMOD, would require a good characterization of survival, which is not currently available in China [6,13].

Although the sensitivity analyses suggest that our results are not unduly sensitive to a number of methodological issues, we have no way of fully validating the burden estimates. Jensen et al. sought to validate their use of MI ratios to estimate burden, later taken up by IARC, by applying the modeled MI ratios for the European Community to mortality data from Scotland and Denmark and comparing predicted with observed cancer incidence for these two regions [8]. Data from Scotland and Denmark were included in the MI calculations, which would tend to reduce the difference between observed and predicted incidence. We applied a similar approach using two registries, Shangahi and Qidong. The percentage variation of estimated from observed incidence rates in these registries – 2.3% to 9.5% depending on sex and registry – was similar to that in the European registries – 1.9% to 7.9%. Had the registries been a representative sample from a larger whole, we could have used cross-validation; however, the registries were not representative and we used all available. Following a reviewer’s suggestion, future work could use simulation to compare several methods for burden calculation. This would allow the simulation of cancer incidence and mortality under different missingness mechanisms, or for bias in the selection of cancer registries, and then assess the degree of bias from the different methods relative to the hypothesized “truth”.

There are several other models available to model incidence and mortality. We recently developed a generalized linear model where mortality has a binomial distribution conditional on the sum of mortality and incidence [14] (see Additional file 2). It would be useful to adapt this model using a smooth function on the logit scale, with the odds ratio providing an estimate of the MI ratio. In related work, Clèries et al. predicted incidence based on a model for the difference between incidence and mortality rates [15]. Their model can be interpreted as modeling survival by the predicted difference divided by the incidence rate. However, survival does not in general appear to be inversely proportional to the incidence rate, so the age-specific intercept would need to adjust for changes in incidence.

While the main aim of this analysis was to produce a probably more accurate estimate of overall cancer burden in China and the burden of major types of cancer, the MI ratios may also be of interest. The observation that the MI ratios were generally higher in rural than in urban areas suggests there is an important difference in resources for cancer control between these areas in China (see Additional file 4: Table S3). They might also indicate a greater level of under-ascertainment of incident cancers (or over-ascertainment of deaths) by rural than urban registries, particularly given the greater prevalence of MI ratios of 1 in rural areas (which means that the recorded number of deaths was equal to or greater than the number of registered cases).

Although these national estimates are only synthetic substitutes for the true cancer incidence in China and there are uncertainties about these estimates, they provide important information for cancer control in China. Based on our estimates, there were more than a half million (541,600) new diagnoses of lung cancer in China in 2005. The number of lung cancer diagnoses has been predicted to increase in the future due to the aging population, high smoking rates and rising tobacco consumption, especially in younger generation, in China <http://global.tobaccofreekids.org/files/pdfs/reports_articles/2007%20China%20MOH%20Tobacco%20Control%20Report.pdf>. It is well established that tobacco smoking causes lung cancer and many other cancers (including stomach, esophagus and liver cancer). Thus, tobacco control is of particular importance in China and should be considered a high priority in national cancer control plan. Evidence in tobacco control from other countries indicated that healthcare professionals have a critical role to play in setting an example for the general public; for example, a high quit rate among doctors in the United Kingdom had a major impact in reducing lung cancer rate. In the shorter term, their advice encouraging smoking cessation has been shown to be effective in persuading older adults to quit leading to a significant reduction in lung cancer rates. In the longer term, discouraging younger people from initiating smoking is particularly important in China because the smoking rate is rising in the younger generations. Other strategies, including the introduction of anti-smoking legislation and increasing cigarette taxes, have been shown to be effective in tobacco control. Some progress has been made in this regard in China, but we still have a long way from being a smoke-free society.

Prevention is generally preferable to cure especially for cancers with very high fatality, such as cancers of the lung, stomach, esophagus and liver. But it may take another decade to see the benefits of cancer control policies such as tobacco control in China. Therefore, it is equally important to ensure that current and future cancer patients will have adequate access to effective treatment including palliative care. We found a total of 2.96 million new cancers were diagnosed in 2005 in China; it is a huge challenge for the Chinese health system to provide adequate health care for this group of patients. To provide health-care to all these cancer patients, we need an adequate supply of health professionals including skilled cancer surgeons, medical oncologists and cancer nurses, and good access to radiation therapy and cytotoxic drugs and supportive care. We believe that priority in treatment should be given to improving the efficiency of existing treatment in a cost-effective way, especially to those living in rural areas and those who cannot afford expensive cancer treatment. All these should be important element of cancer control plan in China.

Early detection of cancer is another important element of cancer control plan which is extremely important in China, as many cancer patients are diagnosed at an advanced stage. Rising awareness of the general public and education of primary and secondary care providers about the early signs of cancer may be the most important and cost-effective way to detect cancer at the earliest time.

## Conclusions

The limited population coverage and unrepresentative distribution of the cancer registries in China will continue to create uncertainty about estimates of the national cancer burden. The uncertainty would limit the use of these national estimates in developing effective cancer control policy in China. Therefore, there is a pressing need to establish a system for more complete and more representative national cancer registration to support effective cancer control. It may take more than a decade to establish such a national cancer registration system. In the absence of such complete and representative data, creative, informed use of limited regional population-based cancer registries data, with appropriate statistical methods, can be a valuable tool for development of cancer control policy in China.

## Competing interests

The authors declare that they have no competing interests.

## Authors’ contributions

WC and BKA selected the research topic. WC, MC and BKA developed the research plan. RZ and SZ retrieved the data and developed the initial analysis. MC developed the analysis methods. BKA and MC reviewed and provided comments on the analysis. WC wrote the initial version of the manuscript. WC and RZ created all tables and figures. XQY revised the manuscript critically and assisted with formatting and language editing. All authors contributed to the revisions of the manuscript. All authors read and approved the final manuscript.

## Pre-publication history

The pre-publication history for this paper can be accessed here:

http://www.biomedcentral.com/1471-2407/13/458/prepub

## Supplementary Material

Additional file 1 Quality control variables for the 32 cancer registries, 2004-2005. Click here for file

Additional file 2 Statistical modeling, R Code for Calculating O-splines, Adapting Code (with permission) from Matt Wand and John Ormerod (2008, 2010) and R code to define the WinBUGS model. Click here for file

Additional file 3 Estimated Numbers of Incident Cancer Cases in 1000s and age adjusted incidence rates per 100 000 by Site in age groups, China 2005. Click here for file

Additional file 4 MI ratios of major cancers in 32 cancer registries, 2004-2005. Click here for file
