# Supplementary material for: Cancer burden in China: a Bayesian approach
Source: BMC Cancer. 2013 Oct 6;13:458. doi: 10.1186/1471-2407-13-458 (PMC3850959; doi:10.1186/1471-2407-13-458)
Supplement: Additional file 1 — Quality control variables for the 32 cancer registries, 2004-2005. [file 1471-2407-13-458-S1.docx]

**Additional file 1**

**Table S1 Quality control variables for the 32 cancer registries, 2004-2005**

| Registry | Urban/rural  location | East/west location | MI | MV (%) | DCO (%) | U_S (%) |
| --- | --- | --- | --- | --- | --- | --- |
| All registries |  |  | 0.66 | 62.06 | 1.50 | 2.59 |
| Urban | Urban |  | 0.62 | 65.38 | 1.80 | 3.07 |
| Rural | Rural |  | 0.77 | 51.13 | 0.52 | 1.15 |
| East |  | East | 0.65 | 60.70 | 1.68 | 2.72 |
| East Urban | Urban | East | 0.62 | 64.72 | 1.99 | 6.29 |
| East Rural | Rural | East | 0.77 | 53.58 | 0.56 | 2.73 |
| West |  | West | 0.67 | 72.05 | 0.19 | 1.59 |
| West Urban | Urban | West | 0.62 | 71.27 | 0.10 | 5.23 |
| West Rural | Rural | West | 0.75 | 73.43 | 0.36 | 1.00 |
| Beijing | Urban | East | 0.60 | 74.03 | 0.16 | 3.34 |
| Cixian | Rural | East | 0.71 | 76.47 | 2.82 | 0.09 |
| Shexian | Rural | East | 0.80 | 81.41 | 0.28 | 0.14 |
| Yangcheng | Rural | West | 0.64 | 90.74 | 0.00 | 0.19 |
| Shenyang | Urban | East | 0.80 | 77.71 | 0.38 | 4.17 |
| Dalian | Urban | East | 0.61 | 72.22 | 1.32 | 1.48 |
| An'shan | Urban | East | 0.61 | 64.73 | 1.52 | 1.42 |
| Benxi | Urban | East | 0.65 | 54.66 | 0.05 | 4.93 |
| Shanghai | Urban | East | 0.64 | 66.14 | 4.69 | 2.91 |
| Jintan | Rural | East | 0.84 | - | 0.00 | 3.03 |
| Haimen | Rural | East | 0.76 | 44.64 | 0.00 | 2.73 |
| Qidong | Rural | East | 0.83 | 45.93 | 0.05 | 0.40 |
| Huaian District,Huai'an | Urban | East | 0.84 | 39.21 | 0.00 | 0.73 |
| Jianhu | Rural | East | 0.76 | 53.74 | 0.00 | 1.06 |
| Dafeng | Rural | East | 0.83 | 33.36 | 1.22 | 1.97 |
| Yangzhong | Rural | East | 0.84 | 62.34 | 0.05 | 0.38 |
| Taixing | Rural | East | 0.68 | 53.24 | 0.00 | 0.28 |
| Hangzhou | Urban | East | 0.56 | 74.01 | 0.36 | 3.45 |
| Jiaxing | Urban | East | 0.58 | 59.17 | 11.43 | 1.56 |
| Jiashan | Rural | East | 0.74 | 67.93 | 0.14 | 0.96 |
| Haining | Rural | East | 0.70 | 50.67 | 0.00 | 1.95 |
| Maanshan | Urban | West | 0.66 | 72.58 | 0.06 | 2.08 |
| Changle | Rural | East | 0.65 | 57.28 | 0.04 | 1.98 |
| Linqu | Rural | East | 0.87 | 42.37 | 0.00 | 0.03 |
| Feicheng | Rural | East | 0.83 | 65.84 | 3.33 | 0.71 |
| Linzhou | Rural | West | 0.78 | 70.03 | 0.65 | 0.49 |
| Wuhan | Urban | West | 0.62 | 71.16 | 0.10 | 2.35 |
| Guangzhou | Urban | East | 0.53 | 50.04 | 2.86 | 4.39 |
| Sihui | Rural | East | 0.64 | 35.42 | 0.88 | 1.50 |
| Zhongshan | Rural | East | 0.71 | 74.49 | 1.03 | 3.07 |
| Fusui | Rural | West | 0.81 | 21.80 | 0.86 | 0.48 |
| Yanting | Rural | West | 0.78 | 79.02 | 0.20 | 0.01 |

MI: mortality to incidence ratio;

MV%: percentage of morphological verified

DCO%: percentage of death certification only;

U_S%: percentage of unspecified cancer sites
