# Supplementary material for: Cancer burden in China: a Bayesian approach
Source: BMC Cancer. 2013 Oct 6;13:458. doi: 10.1186/1471-2407-13-458 (PMC3850959; doi:10.1186/1471-2407-13-458)
Supplement: Additional file 2 — Statistical modeling, R Code for Calculating O-splines, Adapting Code (with permission) from Matt Wand and John Ormerod (2008, 2010) and R code to define the WinBUGS model. [file 1471-2407-13-458-S2.docx]

**Additional file 2**

**Statistical properties of the proposed model compared with the model by Jensen et al**

Let us assume that the number of deaths M and number of incident cases I are independent and are both Poisson distributed with means E(M) and E(I), respectively. This follows our model formulation and is, by our understanding, the baseline formulation used by Jensen et al (1990) as cited in the paper.

*As a useful simplification for our model, given the sum N=M+I, the distribution for M is binomial with probability p and total number of trials N*. Then the maximum likelihood estimator of the odds ratio ψ=p/(1-p) is M/I, and the variance for the estimator for log(ψ) is estimated by 1/M+1/I.

In contrast, Jensen et al propose a different estimator, assuming that M is Poisson with mean φI, where an estimator for φ is M/I, while the variance for the estimator for log(φ) is estimated by 1/M. This essentially ignores any variability in the incidence I.

We make two observations here. First, the estimators for the MIR are the same for both models. As a consequence, comparable regression model formulations are expected to give similar point predictions. Second, the variance estimator for the model used by Jensen et al (1990) under-estimates the “true” variance.

To investigate how the under-estimated variance affects coverage, we simulated for E(N)=E(M)+E(I) in (10,100,1000) combined with an MI ratio in (0.2,0.4,0.6,0.8,1.0). For each combination, we simulated 10,000 times for M and I and then calculated the coverage for estimates of ψ and φ using the binom.test() and poisson.test() functions in R, respectively (see Table). The coverage under the binomial test for ψ was slightly greater than 0.95, with better coverage for larger N. This is consistent with the properties of the Clopper-Pearson test used in binom.test(). In contrast, the Poisson test tended to lead to under-coverage when the MIR was closer to 1.

Table X1: Simulated coverage of the model based on the binomial (as proposed) and Poisson (as per Jensen et al 1990) test distributions, by MI ratio and expected sum for mortality and incidence..

|  |  | MI ratio | | | | |
| --- | --- | --- | --- | --- | --- | --- |
| Test | E(N) | 0.2 | 0.4 | 0.6 | 0.8 | 1.0 |
| Binomial (ψ) | 50 | 0.9682 | 0.9657 | 0.9665 | 0.9637 | 0.9647 |
| (proposed) | 100 | 0.9608 | 0.9602 | 0.9621 | 0.9595 | 0.9606 |
|  | 1000 | 0.9558 | 0.956 | 0.9520 | 0.9532 | 0.9507 |
| Poisson (φ) | 50 | 0.9490 | 0.9256 | 0.9019 | 0.8711 | 0.8542 |
| (Jensen et al) | 100 | 0.9421 | 0.9196 | 0.8934 | 0.8675 | 0.8476 |
|  | 1000 | 0.9321 | 0.9105 | 0.8825 | 0.8593 | 0.8349 |

As a final observation, if the coverage is low for the model used by Jensen et al (1990), then any model selection step will tend to be over-fitted.

**R Code for Calculating O-splines, Adapting Code (with permission) from Matt Wand and John Ormerod (2008, 2010)**

require(splines)

os <- function(x,knots,Boundary.knots=range(x),centre=FALSE) {

nx <- names(x); x <- as.vector(x)

nIknots <- length(knots)

B <- splines::bs(x,knots=knots,degree=3, Boundary.knots=Boundary.knots,intercept=TRUE)

Aknots <- sort(c(rep(Boundary.knots,4),knots))

L <- 3*(nIknots+8)

xtilde <- (rep(Aknots,each=3)[-c(1,L-1,L)]+ rep(Aknots,each=3)[-c(1,2,L)])/2

wts <- rep(diff(Aknots),each=3)*rep(c(1,4,1)/6,nIknots+7)

Bdd <- splines::spline.des(Aknots,xtilde, derivs=rep(2,length(xtilde)), outer.ok=TRUE)$design

Omega <- t(Bdd*wts)%*%Bdd

eigOmega <- eigen(Omega)

inds <- 1:(nIknots+2)

UZ <- eigOmega$vectors[,inds]

LZ <- t(t(UZ)/sqrt(eigOmega$values[inds]))

basis <- B%*%LZ

dimnames(basis) <- list(nx,1:ncol(basis))

if (centre) {

centreBasis <- os(centre,knots=if (is.null(knots)) numeric(0) else knots,

Boundary.knots=Boundary.knots, centre=FALSE)

oldAttributes <- attributes(basis)

basis <- t(apply(basis,1,function(x) x-centreBasis))

attributes(basis) <- oldAttributes

}

a <- list(knots=knots,Boundary.knots=Boundary.knots,centre=centre)

attributes(basis) <- c(attributes(basis),a)

class(basis) <- c("os","basis")

return(basis)

}

os(c(0,1,seq(5,85,by=5)), knots=c(45,55,65,75), centre=45)

**R code to define the WinBUGS model.**

After the model is specified, the user defines the following data: nages, which is the number of age groups; ncodes, which is the number of cancer registries; nknots, which is the number of knots; agegroupc, which is a double precision (DP) vector of centred age values; Z, which is a DP matrix defining the splines; yreginc, which is an integer matrix of the registry incidence; yregmort, which is an integer matrix of the registry mortality; popureg, which is an integer matrix of the registry populations; yndsmort, which is an integer vector with the number of NDS mortality; popunds, which is an integer vector of the NDS populations; and popunat, which is an integer vector of the national population. Then the user defines initial values for the fixed effects, the random effects and the precision terms.

require(BRugs)

model <- function() {

for (i in 1:nages) { # index over age

alphamu[i] <- alphafe[1]+alphafe[2]*agegroupc[i]+inprod(alphare[],Z[i,])

betamu[i] <- betafe[1]+betafe[2]*agegroupc[i]+inprod(betare[],Z[i,])

gammamu[i] <- gammafe[1]+gammafe[2]*agegroupc[i]+inprod(gammare[],Z[i,])

}

for (i in 1:2) { # index over fixed effects

alphafe[i] ~ dnorm(0,1.0E-6)

betafe[i] ~ dnorm(0,1.0E-6)

gammafe[i] ~ dnorm(0,1.0E-6)

}

for (i in 1:nknots) { # index over random effects

alphare[i] ~ dnorm(0,taualpha)

betare[i] ~ dnorm(0,taubeta)

gammare[i] ~ dnorm(0,taugamma)

}

for (i in 1:ncodes) { # index over areas

alphacode[i] ~ dnorm(0,taualphacode)

betacode[i] ~ dnorm(0,taubetacode)

}

taualpha ~ dgamma(0.001,0.001)

taubeta ~ dgamma(0.001,0.001)

taugamma ~ dgamma(0.001,0.001)

taualphacode ~ dgamma(0.001,0.001)

taubetacode ~ dgamma(0.001,0.001)

for (i in 1:nages) { # index over age groups

for (j in 1:ncodes) { # index over areas

mureginc[i,j] <- exp(alphamu[i]+alphacode[j])*popureg[i,j]

muregmort[i,j] <- exp(alphamu[i]+alphacode[j]+betamu[i]+betacode[j])*popureg[i,j]

yreginc[i,j] ~ dpois(mureginc[i,j])

yregmort[i,j] ~ dpois(muregmort[i,j])

}

mundsmort[i] <- exp(gammamu[i])*popunds[i]

yndsmort[i] ~ dpois(mundsmort[i])

predmort[i] <- exp(gammamu[i])*popunat[i]

predinc[i] <- exp(-betamu[i])*predmort[i]

}

}

writeModel(model,"model.bug")
