# Supplementary material for: Cancer burden in China: a Bayesian approach
Source: BMC Cancer. 2013 Oct 6;13:458. doi: 10.1186/1471-2407-13-458 (PMC3850959; doi:10.1186/1471-2407-13-458)
Supplement: Additional file 3 — Estimated Numbers of Incident Cancer Cases in 1000s and age adjusted incidence rates per 100 000 by Site in age groups, China 2005. [file 1471-2407-13-458-S3.docx]

**Additional file 3**

**Table S2 Estimated Numbers of Incident Cancer Cases in 1000s and age adjusted incidence rates per 100 000 by Site in age groups, China 2005**

| Site | Cases | | | Incidence (/100 000) | | |
| --- | --- | --- | --- | --- | --- | --- |
|  | 0-35 | 35-64 | 65+ | 0-35 | 35-64 | 65+ |
| Nasopharynx | 2.9(2.1,3.9) | 23.3(20.4,26.7) | 7.5(6.4,8.8) | 0.45(0.34,0.61) | 4.29(3.76,4.91) | 6.35(5.40,7.48) |
| Esophagus | 1.3(1.0,1.7) | 128.1(119.9,137.0) | 147.1(138.7,155.4) | 0.20(0.15,0.27) | 23.54(22.04,25.18) | 124.37(117.26,131.38) |
| Stomach | 6.8(5.7,8.2) | 226.1(214.9,238.0) | 260.3(248.8,273.2) | 1.06(0.89,1.28) | 41.56(39.50,43.74) | 219.99(210.32,230.92) |
| Colorectal | 8.3(6.3,11.2) | 110.9(100.5,122.4) | 115.5(105.7,126.3) | 2.66(2.03,3.61) | 40.83(37.02,45.07) | 187.39(171.46,204.87) |
| liver | 12.3(11.2,13.9) | 235.2(222.7,248.3) | 163.6(154.5,173.6) | 1.92(1.74,2.16) | 43.22(40.93,45.63) | 138.27(130.60,146.73) |
| Pancreas | 0.6(0.5,0.9) | 18.9(16.8,21.2) | 25.8(23.2,28.8) | 0.10(0.07,0.14) | 3.47(3.10,3.91) | 21.77(19.60,24.31) |
| Lung | 6.5(5.7,7.5) | 232.7(221.0,245.7) | 302.8(288.0,318.8) | 1.02(0.88,1.17) | 42.76(40.62,45.15) | 255.90(243.41,269.46) |
| Bone | 4.5(3.4,6.3) | 13.9(11.8,16.5) | 11.0(9.3,13.0) | 0.71(0.53,0.99) | 2.55(2.17,3.03) | 9.27(7.83,10.98) |
| Breast | 9.6(7.1,13.1) | 136.4(119.1,156.5) | 26.5(22.5,31.2) | 1.49(1.10,2.05) | 25.07(21.88,28.77) | 22.38(18.98,26.35) |
| Cervix | 7.8(5.3,11.8) | 52.4(41.8,65.5) | 8.3(6.5,10.6) | 2.49(1.70,3.77) | 19.31(15.39,24.11) | 13.47(10.63,17.21) |
| Ovary | 2.4(1.5,3.9) | 21.0(16.8,26.4) | 6.5(5.1,8.2) | 0.72(0.47,1.18) | 7.69(6.16,9.70) | 11.43(9.03,14.46) |
| Prostate | 0.4(0.2,1.3) | 6.6(4.7,9.4) | 27.5(22.9,33.1) | 0.07(0.02,0.21) | 1.21(0.86,1.73) | 23.28(19.33,28.01) |
| Bladder | 2.0(1.0,5.2) | 24.9(20.2,31.1) | 34.7(30.2,40.1) | 0.63(0.32,1.68) | 9.17(7.43,11.44) | 56.31(49.05,65.13) |
| Brain,CNS | 12.0(9.9,15.0) | 35.6(31.3,40.9) | 18.4(15.9,21.2) | 3.85(3.17,4.81) | 13.12(11.51,15.06) | 29.82(25.74,34.43) |
| Lymphoma | 4.2(3.1,5.8) | 17.7(14.6,21.4) | 11.4(9.3,14.2) | 1.35(1.01,1.86) | 6.51(5.37,7.87) | 18.54(15.14,23.04) |
| All sites | 133.7(124.0,145.3) | 1521.2(1454.6,1593.4) | 1303.8(1250.3,1356.5) | 20.84(19.33,22.64) | 279.59(267.34,292.85) | 1102.09(1056.80,1146.58) |
