# Supplementary material for: Cancer burden in China: a Bayesian approach
Source: BMC Cancer. 2013 Oct 6;13:458. doi: 10.1186/1471-2407-13-458 (PMC3850959; doi:10.1186/1471-2407-13-458)
Supplement: Additional file 4 — MI ratios of major cancers in 32 cancer registries, 2004-2005. [file 1471-2407-13-458-S4.docx]

**Additional file 4**

**Table S3 MI ratios of major cancers in 32 cancer registries, 2004-2005**

|  | **MI ratio (range)** | | |
| --- | --- | --- | --- |
| **Cancer site** | **All areas** | **Urban** | **Rural** |
| **Nasopharynx** | 0.54(0.29,1.50) | 0.51(0.35,1.25) | 0.68(0.29,1.50) |
| **Esophagus** | 0.79(0.62,1.01) | 0.81(0.62,1.01) | 0.78(0.69,1.00) |
| **Stomach** | 0.75(0.59,0.92) | 0.74(0.62,0.92) | 0.75(0.59,0.91) |
| **Liver** | 0.93(0.75,1.58) | 0.92(0.77,1.26) | 0.93(0.75,1.58) |
| **Pancreas** | 0.93(0.64,1.60) | 0.93(0.69,1.60) | 0.93(0.64,1.35) |
| **Lung** | 0.91(0.67,1.40) | 0.92(0.74,1.40) | 0.86(0.67,1.10) |
| **Bone** | 0.78(0.35,2.73) | 0.70(0.37,2.29) | 1.00(0.35,2.73) |
| **Breast** | 0.24(0.16,0.71) | 0.23(0.16,0.68) | 0.39(0.27,0.71) |
| **Bladder** | 0.37(0.18,0.96) | 0.35(0.23,0.53) | 0.54(0.18,0.96) |
| **All sites** | 0.66(0.53,0.87) | 0.62(0.53,0.84) | 0.77(0.64,0.87) |
